# A variety test platform for the standardization and data quality improvement of crop variety tests

**DOI:** 10.3389/fpls.2023.1077196

**Published:** 2023-01-24

**Authors:** Feng Yang, Zhongqiang Liu, Yuxi Wang, Xiaofeng Wang, Qiusi Zhang, Yanyun Han, Xiangyu Zhao, Shouhui Pan, Shuo Yang, Shufeng Wang, Qi Zhang, Jun Qiu, Kaiyi Wang

**Affiliations:** ^1^ Information Technology Research Center, Beijing Academy of Agriculture and Forestry Sciences, Beijing, China; ^2^ National Agro-Tech Extension and Service Center, Beijing, China; ^3^ Key Laboratory of Agri-informatics, Ministry of Agriculture, Beijing, China; ^4^ National Engineering Research Center for Information Technology in Agriculture, Beijing, China; ^5^ AgChip Science and Technology (Beijing) Co., Ltd., Beijing, China

**Keywords:** crop variety test, trait data, standardization, data quality control, statistical analysis

## Abstract

Variety testing is an indispensable and essential step in the process of creating new improved varieties from breeding to adoption. The performance of the varieties can be compared and evaluated based on multi-trait data from multi-location variety tests in multiple years. Although high-throughput phenotypic platforms have been used for observing some specific traits, manual phenotyping is still widely used. The efficient management of large amounts of data is still a significant problem for crop variety testing. This study reports a variety test platform (VTP) that was created to manage the whole workflow for the standardization and data quality improvement of crop variety testing. Through the VTP, the phenotype data of varieties can be integrated and reused based on standardized data elements and datasets. Moreover, the information support and automated functions for the whole testing workflow help users conduct tests efficiently through a series of functions such as test design, data acquisition and processing, and statistical analyses. The VTP has been applied to regional variety tests covering more than seven thousand locations across the whole country, and then a standardized and authoritative phenotypic database covering five crops has been generated. In addition, the VTP can be deployed on either privately or publicly available high-performance computing nodes so that test management and data analysis can be conveniently done using a web-based interface or mobile application. In this way, the system can provide variety test management services to more small and medium-sized breeding organizations, and ensures the mutual independence and security of test data. The application of VTP shows that the platform can make variety testing more efficient and can be used to generate a reliable database suitable for meta-analysis in multi-omics breeding and variety development projects.

## 1 Introduction

Innovation in crop variety is essential for increased grain production. In most countries, new and improved crop varieties have to undergo tests, registration, and approval before their benefits can be realized (Setimela et al., 2010; Yan, 2021). Regional variety tests are often conducted to obtain agronomic information, such as agronomic characteristics, tolerance to disease, and quality. These data are vital for the evaluation of new varieties and can support the decision process for the selection of suitable new varieties for a target area. Therefore, it is critical to ensure the quality of the tests through accurate data.

The variety test process generally includes test design and application, data acquisition, data processing, and analysis. Human errors may occur in each part of the process, such as sowing date in wrong format and plant height exceeding the range, which will affect the accuracy of the test data. In particular, variety tests are generally conducted across different locations and over several years. There will inevitably be differences in the experience of testing personnel at each location, which may also lead to inconsistencies in the data. For example, disease is recorded as incidence rate in some locations and as disease grade in other locations. The implementation quality of each process step will affect the final test results, so standardized test processes are vital to ensure test quality.

The development of high-throughput phenotypic technology has facilitated the collection of variety testing data significantly (Yang et al., 2020). However, due to the lack of effective tools and management systems, in many cases test data are recorded on paper or in Excel spreadsheets (Köhl and Gremmels, 2015; Jung et al., 2021). Moreover, variety phenotyping datasets are often very heterogeneous in terms of types, quantity, quality, formats and sources (field, laboratory, etc.) (Leonelli et al., 2017; Papoutsoglou et al., 2020). Additionally, there is usually a lack of data standardization in terms of syntax, semantics, and structure (Brown et al., 2020). Therefore, test data management and the utilization of the data’s value are time-consuming and laborious, and this will seriously affect the variety evaluation efficiency and accuracy.

In order to help plant breeders manage and analyze data, and track genealogies, plant breeding software has been developed and applied. Examples of such software include AGROBASE (Agronomix Software, 2022), Genovix (Agronomix Software, 2022), PRISM (Central Software Solutions, 2022), BMS (Integrated Breeding Platform, 2019), GoldenSeed (Han et al., 2018; Zhao et al., 2022), and BIMS (Jung et al., 2021). Moreover, Laboratory Information Management Systems (LIMS) are also used in plant breeding data management, and have been found conducive to the management of genotype data in a moderately high throughput genotyping laboratory (Jayashree et al., 2006). Additionally, some software has been developed for high-throughput phenotypic data management. Nieuwland et al. introduced a laboratory management system (Phytotracker) (Nieuwland et al., 2012), which was designed specifically to organize and track plasmids, seeds, and plant growth. Although breeding management software, LIMS, and phenotypic software are also applicable for some variety characteristic tests, they cannot meet the whole workflow management requirements of regional new variety tests. The regional tests of varieties mainly focus on field performance and the value of cultivation and use of varieties. Next-generation phenotyping generates significantly more data than in the past and requires novel data management and access techniques and storage systems, increased use of ontologies to facilitate data integration, and new statistical tools (Cobb et al., 2013). Therefore, it is essential to build a variety testing management system for the whole workflow, which could be used for the efficient management of test process and trait data.

In this study, we demonstrate a variety test management platform to improve test management efficiency and data quality. The proposed platform can be employed for the whole workflow of variety tests, test process standardization, test efficiency improvement, and promoting the sharing and utilization of test data. Therefore, there are three aspects that have to be addressed. First, some data element standards and basic datasets for plant phenotypic traits and descriptions are constructed to ensure the interoperability and integration of phenotypic data that obtained from different locations. Second, automatic tools (such as trial design, data processing, data analysis, etc.) are required to manage the whole testing workflow. Third, the regional variety test management platform needs to be based on Software-as-a-Service (SaaS) architecture to ensure scalability.

## 2 Materials and methods

### 2.1 SaaS-based system architecture

**Figure 1:**
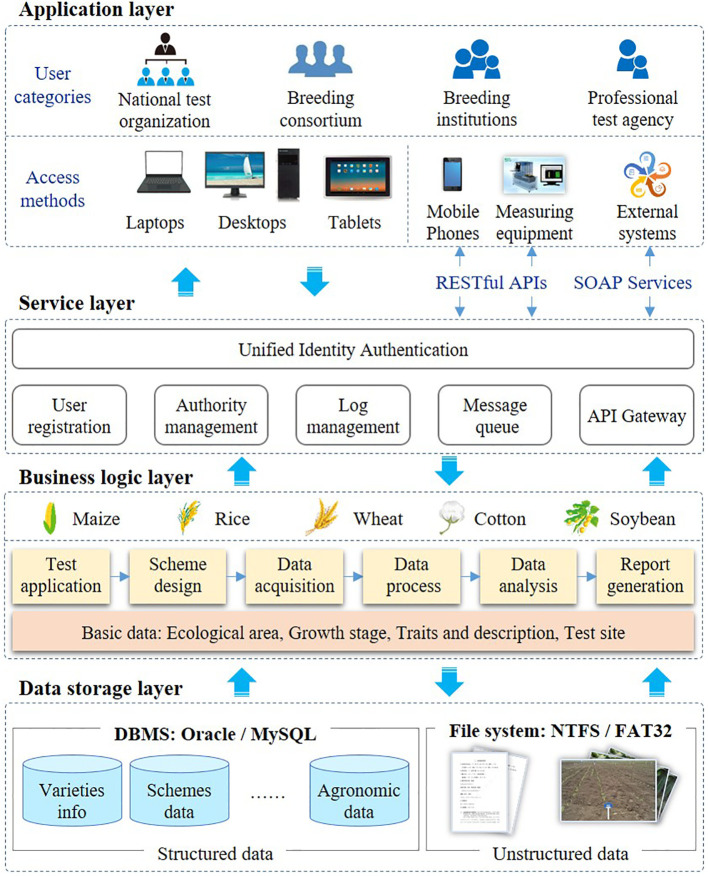
SaaS-based Framework of VTP.

The proposed variety testing platform (VTP) was implemented as a SaaS architecture because of the requirement for multi-tenancy, efficiency, configurability, scalability etc. A SaaS cloud implementation makes the system exceptionally scalable and can provide customized on-demand services for more users. Moreover, it can reduce the requirements on users’ IT resources and technical personnel costs. SaaS has received significant attention from software providers and users as a software delivery model (Aleem et al., 2021), and most of existing companies are transferring their business into a SaaS model to (Almorsy et al., 2014). In particular, the proposed SaaS-based VTP allows users pay more attention to their business, and allows the provision of services to large numbers of small and medium-sized breeding enterprises and research institutions. As shown in **Figure 1**, the VTP is designed in a flexible four-tiered architecture, which is divided into the application layer, the service layer, the business logic layer, and the data storage layer.

Based on SaaS architecture, VTP can provide services for different categories of users, such as national test organization, breeding consortium, breeding institutions and professional test agency. The VTP uses a web-based architecture, which allows the platform to be available throughout the Internet to any terminal device (e.g., notebook computer, tablet, or smartphone) equipped with a web browser. The VTP supports various data interfaces, such as Web Services and a RESTful API. With the wide application of phenotypic devices, more and more observation data will be uploaded from phenotypic devices to VTP through data interfaces. As shown in **Figure 1**, these data interfaces are mainly used for data collection and data sharing. The data of traits recorded through APP or measuring equipment can be transferred to VTP through RESTful. Trait data is shared mainly through Web service. Some statistical data, such as the number of varieties and test sites, need to be shared with the seed industry big data system. In addition, the trait data of variety test should be shared with the variety approval system.

The service layer is responsible for providing reusable services, such as unified identity authentication, log management, message queue, etc. Data access is an essential aspect that needs to be considered for SaaS systems (Mezni et al., 2018). Functional and data access management were designed in the VTP. Through the configuration of functional and data access, the users of the VTP can not only enjoy the security of their business data, but can also share basic data sets such as traits and descriptions. Users at each test location can only see the data of the varieties they test and even within the same location, the permissions of different roles are independent of each other. The main functionalities of the VTP include variety review, trial design, data acquisition, statistical analysis, and test report generation. In variety testing, trial design, data acquisition and data analysis are the three most important steps, and are also the most laborious and time-consuming. Meanwhile, these three steps are also the ones most likely to be affected by human errors. Therefore, a series of data standards are proposed for testing process management and data quality control. Additionally, some standardized data elements (e.g., traits, locations, ecological zones, etc.) are essential for all functionalities in the VTP. In different programs, these basic data can be configured according to actual needs. By developing some automatic tools, human errors can be avoided and the efficiency of test design, data acquisition and data analysis can be improved.

In the data storage layer, multi-tenant shared database architecture is adopted. Oracle database technology is used as the data storage and management database, and Redis (https://redis.io/) as the cache database. MySQL can also be used to replace the Oracle database. Unstructured data, such as pictures and documents, are stored on disk in the form of files.

The VTP was programmed with Eclipse IDE (https://www.eclipse.org) and Java. The Spring MVC framework is used for front-end control, while Hibernate is used for data access. The graphical user interface is implemented in Kendo UI (https://www.kendouicn.com). The servlet container is Apache Tomcat (https://tomcat.apache.org/). Nginx (http://nginx.org) is used as reverse proxy server.

### 2.2 Business process standardization

**Figure 2:**
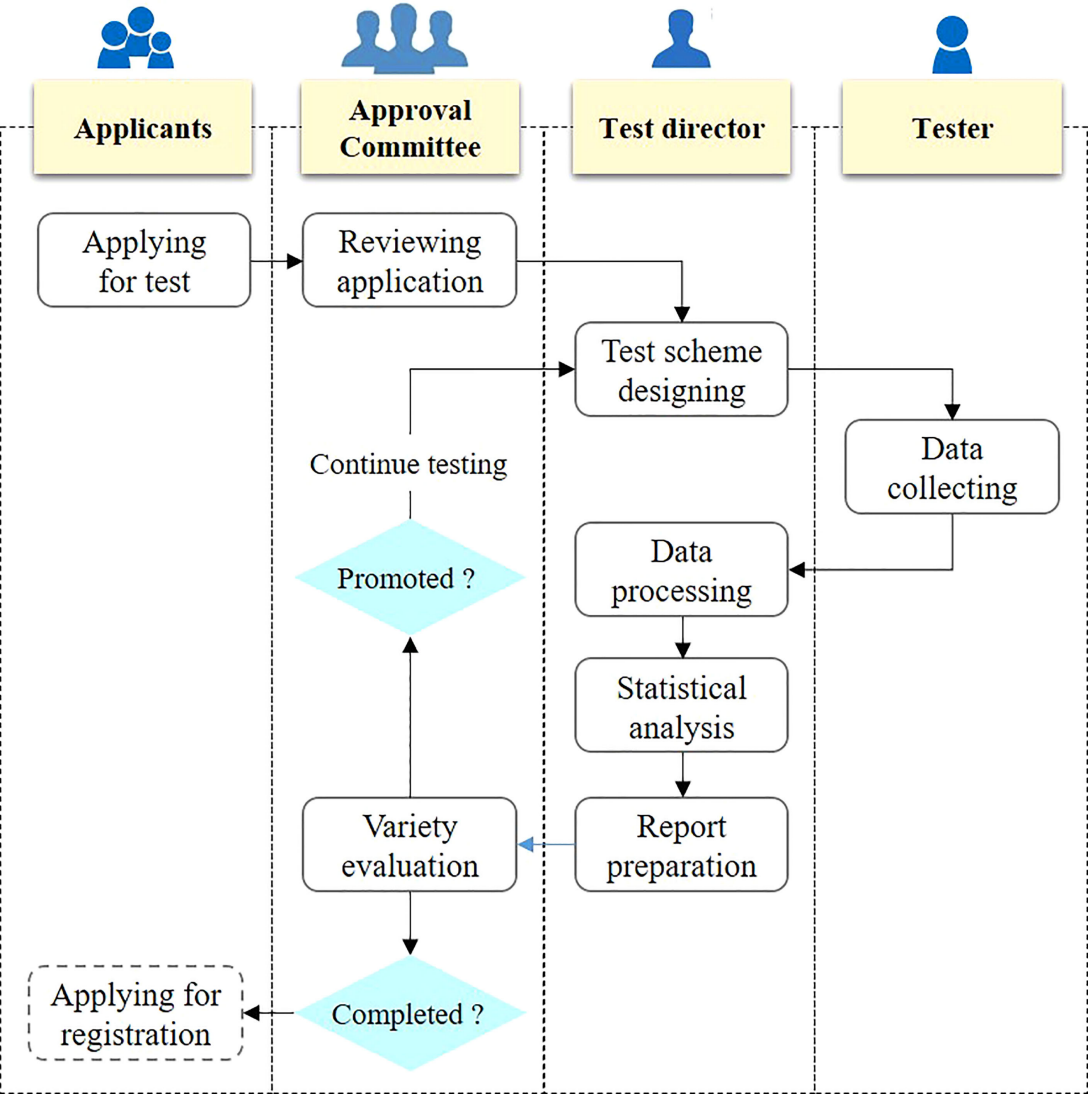
Business process of crop variety test.

The variety test is usually carried out in multiple test sites for many years. Take the maize variety test in Huang Huai Hai ecological zone as an example, it needs to be carried out in about 40 test sites for two to three years. Different test sites have different people responsible for collecting test data, and the lack of uniform operation specifications may lead to inconsistent test quality. The standardization of variety test process will help to standardize the test process and improve the test quality. According to the process of variety test, variety test can be divided into variety test application, application review, test scheme design, test data collection, test data processing, test data analysis, and test report preparation, as shown in **Figure 2**. First of all, the participants shall submit the application for participation according to the specification, so as to ensure the consistency of the information of the tested varieties. Second, the variety approval committee shall review the varieties to be tested to ensure the rationality of the application. Third, the director of the variety test is responsible for the test scheme design, and this director is responsible for the test of the entire ecological zone, so as to ensure the consistency of the test design of each test site. Fourth, each test site shall carry out the test according to the test plan and collect the test data. Fifth, the test data shall be processed and statistically analyzed by the test director. Sixth, the test director shall prepare the test report and submit it to the variety approval committee. Seventh, the variety committee shall carry out evaluation according to the variety evaluation criteria.

### 2.3 Data element standards and basic data sets

The main task of variety testing is to observe and record the characteristics of varieties from multi-location and multi-year tests. However, due to a lack of data element standards, the trait records from different testing locations may have differences in trait names, units, etc. These differences will hinder data integration and the reuse of different test points for analysis. Consequently, data element standards were established based on relevant variety test standards, a controlled vocabulary, and international information standards, such as the Regulations for the Variety Tests and Informatization of Field Crop–Maize (NY/T 1209–2020), Regulations for the Regional Tests of Crop Varieties–Soybean (NY/T 1299–2014), Technical Procedures for Rice Variety Regional Test (NY/T 1300–2007), Technical Procedures for Wheat Variety Regional Test (NY/T 1301–2007), and the Technical Procedures for Cotton Variety Regional Test (NY/T 1302–2007), MIAPPE (Ćwiek-Kupczyńska et al., 2016).

**Figure 3:**
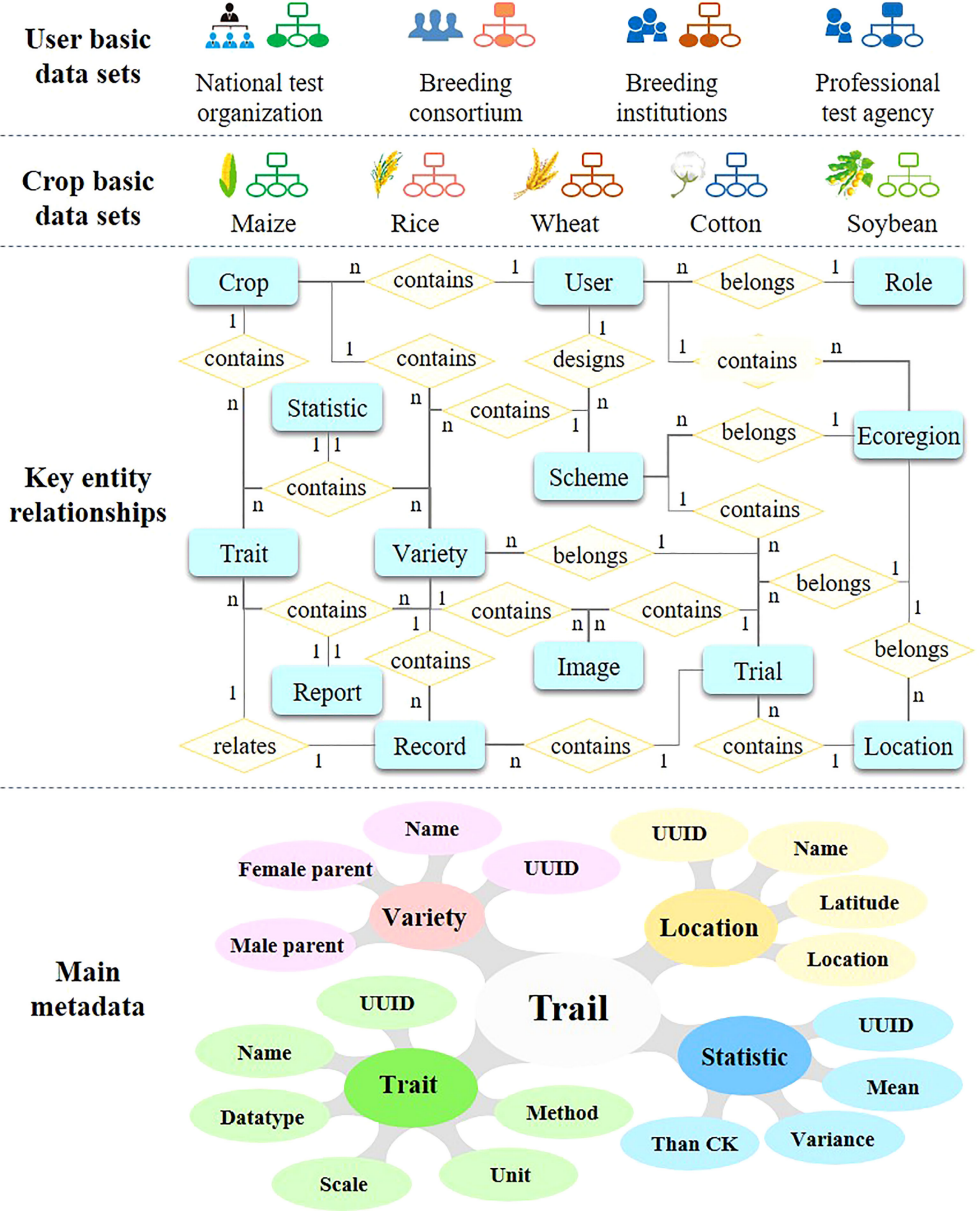
Main data organization mode of VTP.

As shown in **Figure 3**, the main entities of variety testing data involve Trait, Trial, Record, and Statistic. Each data entity is further described as multiple attributes. For instance, the trait is consist of name, description, unit of measure, data type, method, etc. Moreover, more than 13 entities and the entity relationship model in VTP are identified. On this basis, the basic data set and controlled vocabulary of each crop can be extracted. In addition, according to the needs of actual users, the basic data set and controlled vocabulary that are suitable for them can be filtered.

Furthermore, based on these data element standards, some common basic elements were built as an important support tool for VTPs operation. The VTP must be able to handle the flexible configuration of test characteristics for different programs. For instance, taste and quality characteristics are more pertinent when evaluating fresh maize varieties. For different crops, the characteristics of variety testing may also be different. Thus, in different testing schemes, the user does not necessarily need to establish new basic elements; they only need to select the basic elements they require from a common set of such elements. In this way, the same data description can be applied to all test locations to ensure the consistency of test data, even under different testing programs. Different crop varieties have different traits. For example, maize needs to record the number of rows per ear, ear diameter, etc., while rice and wheat do not record these traits. In order to be compatible with different crop varieties at the same time, VTP establishes a set of traits for each crop variety, and each crop variety shares the metadata of traits.

Accordingly, based on the data element standards and the basic elements, the trait records in the VTP are stored in the form of entity-attribute-value (EAV). This data storage model is especially suitable for so-called sparse and heterogeneous data, which refers to cases where the number of attributes of an object is small compared to the number of attributes that could be measured, and tends to change during a program (Dinu and Nadkarni, 2007; Köhl and Gremmels, 2015). These data are stored in a central data repository with a highly standardized data storage structure for processing and sharing.

### 2.4 Experiment scheme design

Variety test design mainly refers to the grouping and field layout of the varieties to be tested, which is an essential premise for reliability of the test results. In China, the regional tests of varieties are generally arranged in randomized complete blocks and repeated three times. In each regional test, there are no more than 16 varieties in the same block. Tests with few varieties are arranged by interval contrast design without repetition. Generally, one or two approved main varieties are set for each district group as the checked variety (CK). However, due to the large number of varieties tested every year, factors such as a variety’s participation in other tests need to be taken into account during the test design process. Some test programs also require the anonymization of varieties to ensure test fairness. Consequently, the experimental design process is time-consuming and laborious, and thus easily susceptible to human error.

**Figure 4:**
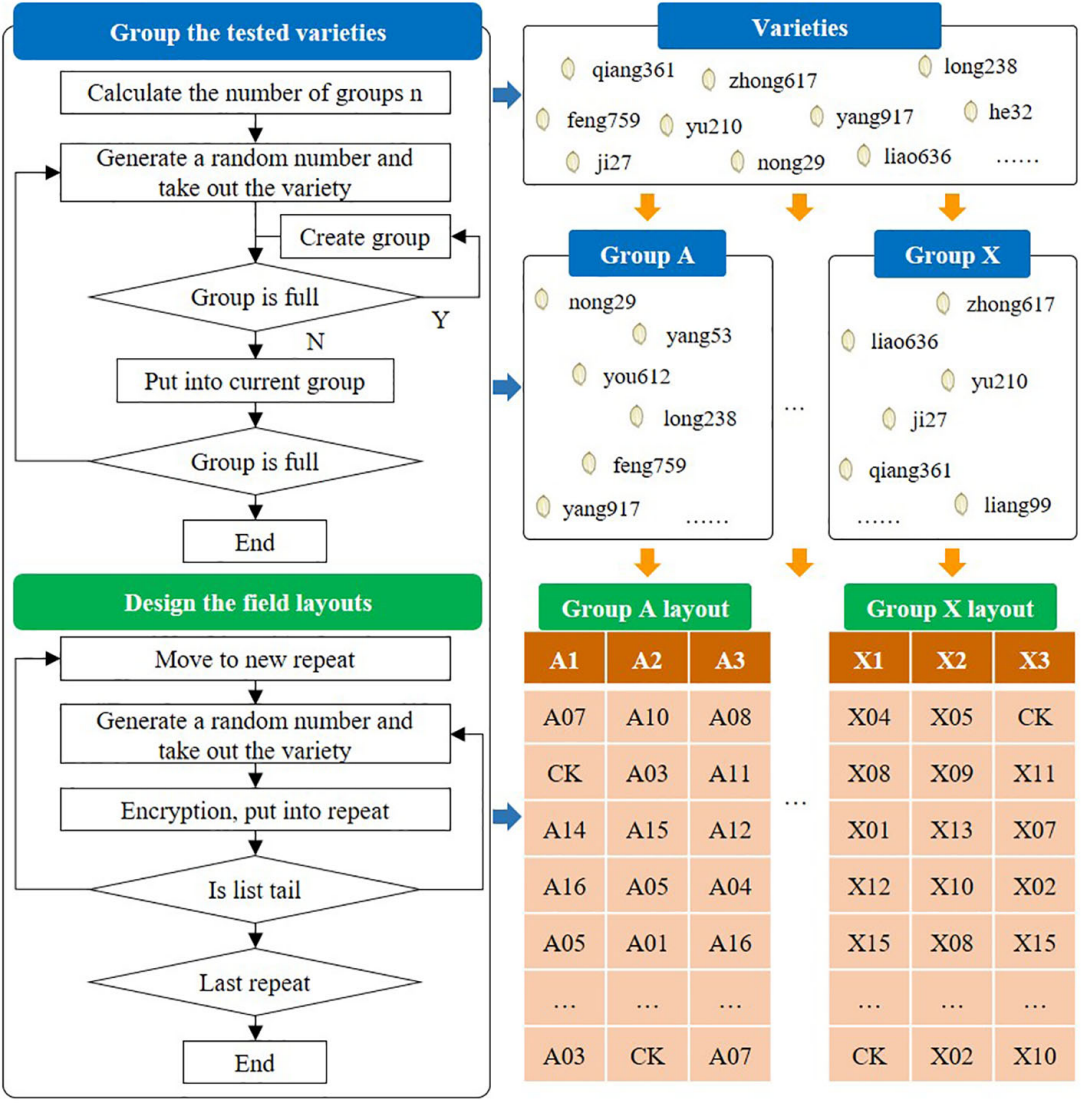
Processes of variety test design.

In order to facilitate test designers, it is necessary to provide more functions for automated design. First, the varieties participating in the test are counted, the number of varieties participating in a group is defined, and then the system will automatically calculate the number of groups to be divided. Second, a variety is randomly selected from the list of grouped varieties and is placed in an unfilled group. If the group is full, the variety is placed in the next available group. The above steps are repeated until there are no more ungrouped varieties and variety grouping has been completed. Next, each group is associated with a preset test location and this is defined as a test. For the three repeated tests, the field layout needs to be designed, as shown in **Figure 4**.

### 2.5 Data acquisition and quality control

**Figure 5:**
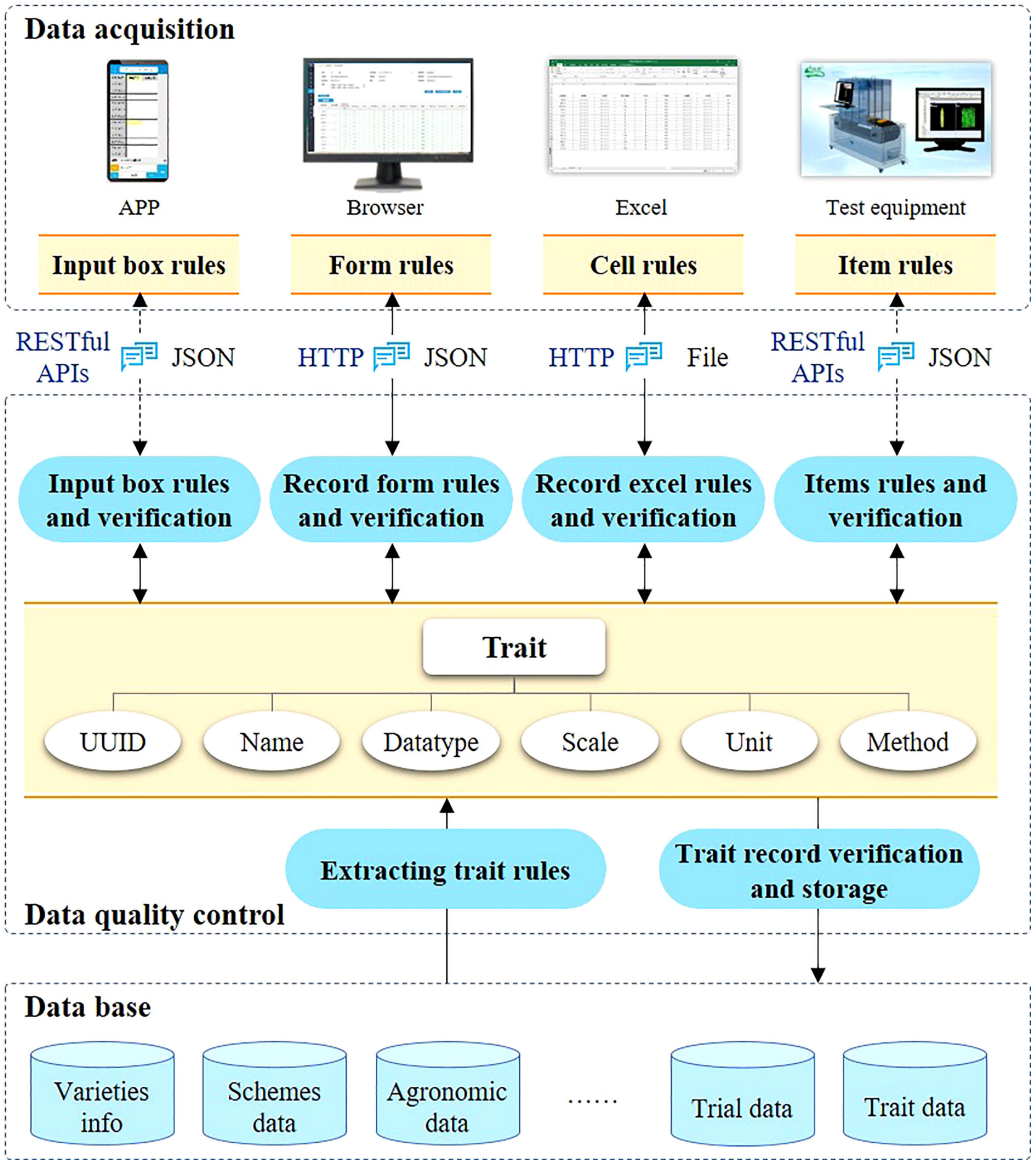
Data acquisition and quality control.

Accurate and reliable trait observation data is the basis for constructing a high-quality phenotypic database and multi-omics research. The data flow chart of variety testing is shown in **Figure 5**. Initially, the testers observe characteristics and submit them to the data management system. Then, the test director, who is in charge of the test, processes and analyzes the data. Finally, experts of the variety approval committee will evaluate and select the upgraded varieties according to the evaluation criterion. At present, phenotypic data includes mainly field observation records and laboratory measurement records. In the processes of data entry and conversion, it is likely that errors are introduced due to improper human operation or low proficiency of the users.

The quality control of test data is mainly through manual review or assisted by the computer. Constraint rules are used to restrict the input data, and possible errors are identified and evaluated to improve the accuracy and availability of data. The traits to be observed and the recording tools to be used are also different at different crop growth stages. For instance, the color of a leaf sheath at seedling stage needs to be observed and recorded in the field conditions, so a mobile device application may be more convenient for this purpose. In the seed measure stage, an Excel file including the ear row number, ear diameter, and other information can be directly obtained through the observation equipment. It often more convenient to import Excel files to the system directly.

Therefore, the VTP provides a variety of data input methods. First, using the mobile application, users can log in to their own account and instantly obtain the test task and the traits to be observed and recorded through direct data entry. Second, users can fill in an online spreadsheet-like form generated according to the standard properties to be recorded. Third, users can download notebooks, which are again generated according to the standard properties to be recorded. Users cannot edit the header of notebooks, but only fill in the value of the corresponding traits. In addition, the VTP provides a variety of interfaces to support data pushing from high-throughput phenotypic observation devices. These data are preliminarily verified according to the rules shown in **Table 1**, for the validity of values in fields such as range and format.

**Table 1:** Example of preliminary data verification rules.

Checking type	Constraint	Abnormal value example
Range	Numerical type: Min ≤ *x* ≤ Max	Maize plant height: 5 meters, generally lies in the range 0.5 ~ 5 meters
	Enumeration type: *x* in collection	Maize plant type: Flat and compact, semi-compact
Format	Date type: YYYY-MM-dd	Sowing date: 9021.6.3
	Numerical type: two decimal places, ^[0-9]+\[0-9]{2}$	Plot yield: 16.7.8
Consistency	Numerical type: y=a*x*+*b*	Y=25.3 kg, *x*=49.2 g *x*: 100 grain weight, y: plot yield
Statistical characteristic	CV: N ≤ y ≤M	Plot yield CV: 25.30

At the same time, after the data are stored in the database, they can also be verified through consistency and statistical characteristics.

### 2.6 Data statistical analysis

The purpose of variety testing is to compare and evaluate the varieties and locations through the statistical analysis of the test data, which mainly includes data quality analysis, test location evaluation, and variety evaluation. This kind of analysis requires specific professional skills and is time-consuming and laborious. Therefore, it is essential to provide a set of tools, including common statistical analysis methods, which can be used for the analysis of the outcomes of regional variety trials.

The correct evaluation of varieties in regional trials largely depends on the accurate identification of differences among varieties, and the accuracy of regional trials is the main factor affecting the accuracy of identification of differences among varieties. CV (coefficient of variation) is usually used to express the accuracy of the test in regional tests (Kong et al., 2001). The test accuracy can be calculated by Eq. (1), where *x_i_* represents the observed value of each variety, *n* represents the number of varieties, $\bar{x}\;$ represents the average value of the test. Generally speaking, if the CV of the field test is less than 10%, the test error is well controlled. Variety comparison precision refers to the minimum difference between varieties that can be identified in regional trials, which can be represented by the relative minimum significant difference (RLSD). Combined with these two indicators, the overall accuracy of the regional trial can be evaluated. In addition, the genetic coefficient of variation (GCV) can be used to evaluate the resolution of test sites.


(1) $$\text{CV}=\sqrt{\sum_{i=1}^{n}{\left(x_{i}-\bar{x}\right)}^{2}/n-1}*100\%/\bar{x}$$

(2) $$Y_{ijk}=\mu +g_{i}+e_{j}+\theta_{ij}+\epsilon_{ijk}$$

Analysis of variance (ANOVA) is usually used to test the significance of the difference between two or more samples. The model of observed value of variety trait can be represented as Eq. (2), where *Y_ijk_* is the *k*th repeated observation value of the *i*th variety in the *j*th location, μ is the overall mean, *g_i_* is the effect of the *i*th variety, *e_j_* is the effect of the *j*th location, *ε_ij_* is the genotype × environment interaction of the *i*th variety in the *j*th location, *ϵ_ijk_* is the residual variation contributed by the *k*th replicate of the *i*th variety in the *j*th location in the kth year. After the one-way ANOVA is completed based on the above model, we can get a conclusion about whether the control variable has a significant impact on the observation variable. If the control variable does have a significant impact on the observation variable, the multiple comparison method should be used to determine the impact of different levels of the control variable on the observation variable. Compared with Duncan, Shortest Significant Ranges, Student-Newman-Keuls, etc., Least Significance Difference (LSD) can quickly sort a group of data from small to large. Therefore, LSD is usually used for multiple comparisons among varieties.

The ultimate purpose of regional trials of crop varieties is to evaluate the production capacity and environmental adaptability of the tested varieties. The true performance of varieties on each pilot site, that is, the true values of various traits are estimated by arithmetic mean. Generally, the production capacity of varieties are evaluated mainly based on the adjacent standard comparison with the control varieties. The adaptability of varieties is mainly evaluated by their stress resistance, and the strategy of one vote veto is adopted for some disease resistance and insect resistance indicators. The algorithm of variety selection for the example of high-yield maize variety selection is shown in **Table 2**. In this case, variety selection is based on multiple characteristics, such as yield, quality, disease and insect resistance, etc. If the result is 1, the variety should be retained for promotion or further test.

**Table 2:** Algorithm of variety evaluation.

Algorithm: variety evaluation
Input: y1: yield increase compared with CK in the first year y2: yield increase compared with CK in the second year y: mean yield increase compared with CK q1: grain bulk density q2: crude starch content (dry basis) q3: crude protein content (dry basis) q4: crude fat content (dry basis) r1: sum of lodging and folding rate r2: proportion of test locations with the sum of lodging and folding rate ≥ 10.0% p: days longer than the growth period of CK d: highly infectious diseases result: evaluation results result=0 conditionl=(y ≥ 5.0% and y1 ≥ 3.0% and y2 ≥3.0% $\text{condition}2\;=\;\left(\text{q}1\geq 720\frac{\text{gL}}{,}\text{ q}2\geq 69.0\%,\text{ q}3\geq 8.0\%,\text{ q}4\geq 3.0\%\right)$ condition3=r1 ≤ 8.0%, r2 ≤ 20% condition4= p ≤ 2.0 days condition5 = d in (Leaf blight, Stem rot, Ear rot, Head smut, Gray spot) If (condition1 and condition2 and condition3 and condition4 and condition5) result = 1 end if return result Output: 0-> eliminate; 1-> promotion

The comprehensive evaluation of varieties is to evaluate the comprehensive production performance of varieties according to their various characteristics. TOPSIS (Hwang et al., 1981) is a sort method approaching ideal solution and a common method in multi-objective decision analysis at present, which has been applied in crop breeding (Wang et al., 2011). The combination of expert scoring method and entropy weight method to improve the calculation method of index weight is more in line with the needs of current crop breeding work (Pan et al., 2018). The steps of using the improved TOPSIS method to evaluate varieties are show in Eq. (3)-(10):


(3) $$w_{j}=\frac{\alpha_{j}\times \beta_{j}}{\sum_{i=1}^{n}\alpha_{j}\times \beta_{j}}$$

(4) $$v_{ij}=y_{ij}\times w_{j}$$

(5) $$V={\left(v_{ij}\right)}_{m\times n}$$

(6) $$S^{+}=\left\{s_{1}^{+},s_{2}^{+},\cdots ,s_{n}^{+}\right\}$$

(7) $$S^{-}=\left\{s_{1}^{-},s_{2}^{-},\cdots ,s_{n}^{-}\right\}$$

(8) $$D_{i}^{+}=\sqrt{\sum_{j=1}^{n}{\left(v_{ij}-s_{j}^{+}\right)}^{2}}$$

(9) $$D_{i}^{-}=\sqrt{\sum_{j=1}^{n}{\left(v_{ij}-s_{j}^{-}\right)}^{2}}$$

(10) $$C_{i}=\frac{D_{i}^{-}}{D_{i}^{+}+D_{i}^{-}}$$

where *w_j_* is the *j*th comprehensive weight of indicators, *α_j_* is the *j*th index weight obtained based on entropy weight method, *β_j_* is the expert scoring value of the *j*th indicator, and n is the number of indicators, *y_ij_* is the standardized value of the *j*th trait of the *i*th variety, *S* ^+^ is the vector of positive ideal variety, *S* ^-^ is the vector of negative ideal variety, $D_{i}^{+}$ is the distance between the *i*th variety and the positive ideal variety S^+^, $D_{i}^{-}$ is the distance between the *i*th variety and the negative ideal variety S^+^, *C_i_* is the relative closeness between each variety and ideal material. So *C_i_* can be used as the comprehensive score of varieties. All varieties are ranked according to C, and then the comprehensive ranking of all varieties to be evaluated can be obtained. In addition, Yan (2014) put forward the idea of combining a genotype’s main effects (G) with genotype-by-environment interaction effects (GE) to form the genotype main effect plus genotype-by-environment interaction effect model (GGE), which is coupled with double mapping. The model is also called environment-centered principal component analysis, and integrates the principal genotype effects into the interaction term for singular value decomposition after subtracting the environmental mean from the original data. The VTP is able to provide a GGE double plot as a visual method for statistical analysis.

## 3 Results

### 3.1 Summary of best practices

The VTP can be used for the whole workflow management of crop variety testing, including test application, trial design, data collection, data analysis, and report generation. The primary users of VTP include testers, variety approval committees, test directors, and test location managers.

**Figure 6:**
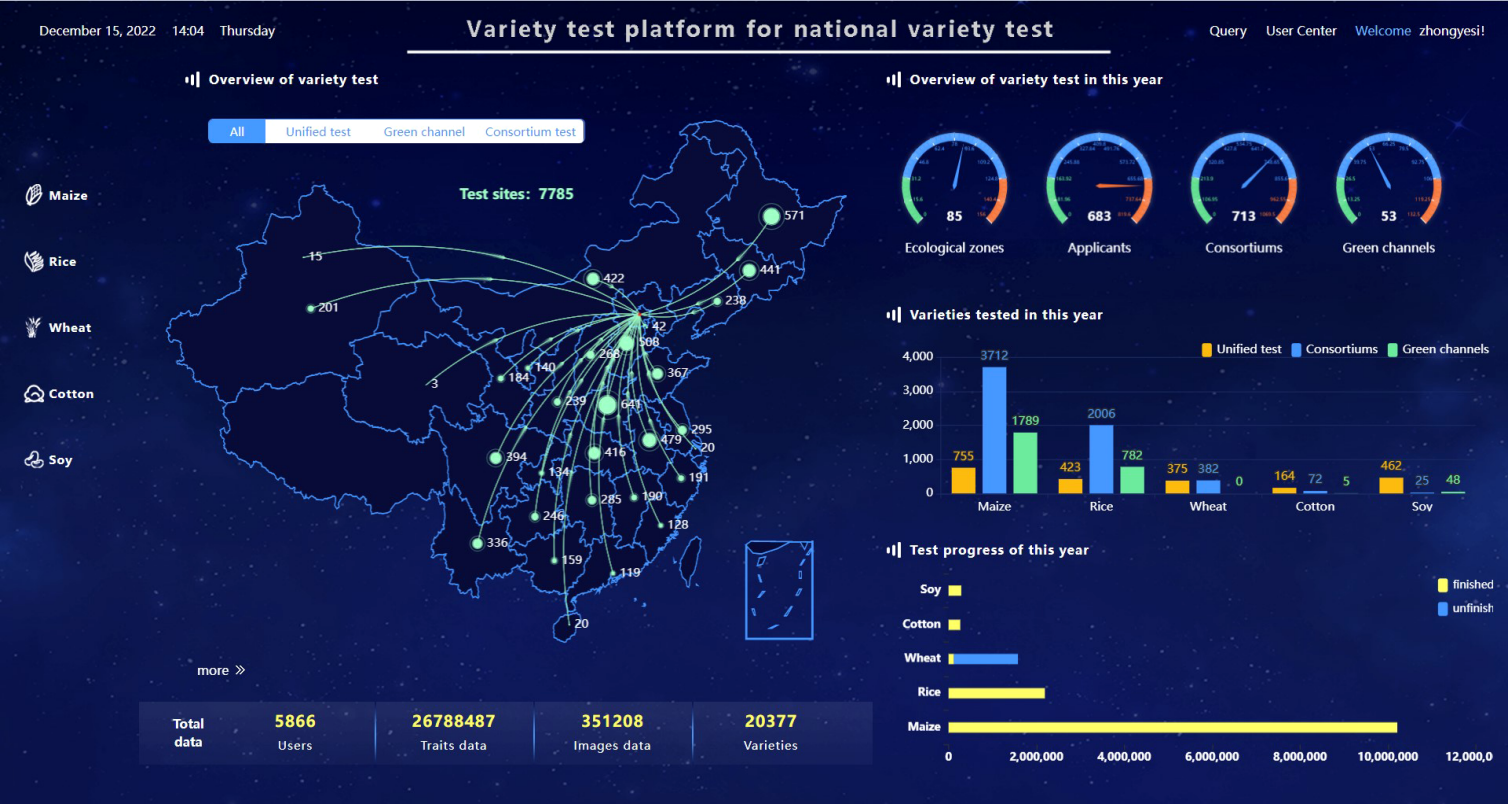
VTP interface for variety test data overview.

The VTP has been employed by the Chinese government to manage the Chinese National Crop Variety Regional Trials, covering maize, rice, wheat, cotton, and soybean since 2017. Since 2020, the VTP has been used to two self-organized testing, consortium and green channel. Now, it provides management services for variety regional testing for more than five thousand users, including all 85 ecological zones of five crops, 20377 varieties, 26788487 trait records and 351208 images (**Figure 6**). There are about six thousand new varieties participating in the regional variety trials every year, which generate eight million agronomic traits data entries, and approximately seventy thousand trial images.

### 3.2 Improving the standardization of the trial process

**Figure 7:**
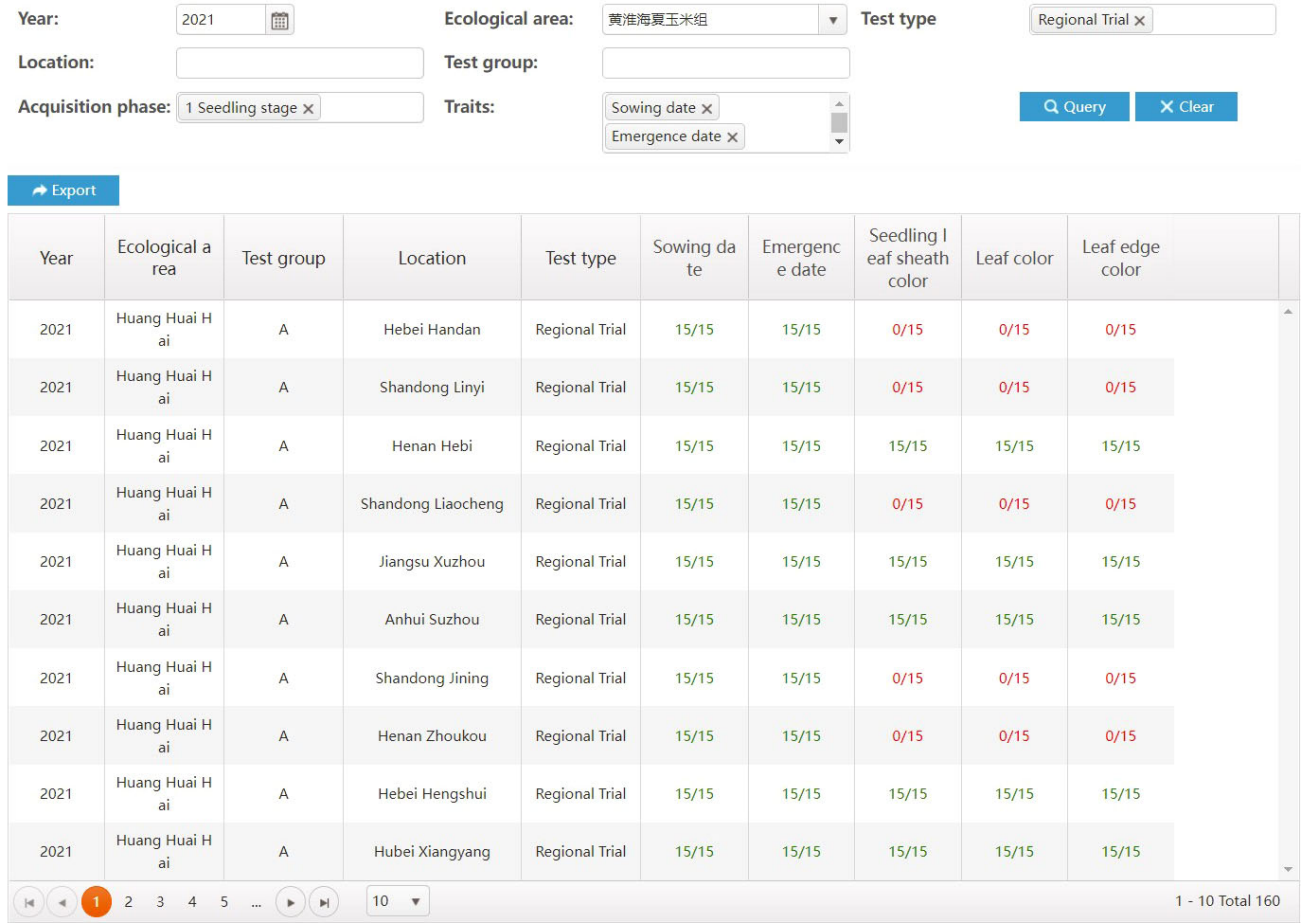
Data collection process.

Reliable and high-quality data come from standardized business processes. Through access control, each type of user can only access the functions and data matching the user’s role. When a user completes their task, the business workflow will move to the next user. For instance, after a test director completes the test design, the task list at each test location will be generated automatically. Then, each user at the test location can see their own tasks, including the varieties to be tested and the characteristics to be observed. Moreover, the director can see in real time how many traits have been collected during the implementation of the test. In **Figure 7**, columns with the form M/N represent show the completion progress of a trait recording task, where M represents the number of samples that have been collected, N represents the target number of samples, green represents a completed collection task, and red represents an uncompleted collection task.

### 3.3 Improving the efficiency of trial management

With the adoption of automatic tools such as trial design, data processing and analysis, and report generation, VTP has significantly improved the efficiency of variety testing management. Some statistics and comparisons on the efficiency of different activities before and after the implementation of VTP are shown in **Table 3**.

**Table 3:** Results of the comparison before VS after the VTP application.

Variety testing activities	Before VTP	With VTP
**Time consumed in checking 100 varieties to be tested**	5 h	3 min
**Time consumed in designing 1 trial**	3 h 30 min	15 min
**Time consumed for processing the data of 16 varieties**	4 h	10 min
**Time consumed in compiling the trial report of 1 ecological zone**	7 h	30 min

There are more than six thousand regional variety testing applications every year. The same variety can only participate in a single test in an ecological zone. Therefore, it is necessary to verify the name, parents, and applicants of varieties to ensure that varieties participate in the test of this ecological zone for the first time. In the original manual methods employed, the reviewers needed to look up each datum one by one in a spreadsheet list. After adopting the system, the variety information verification is carried out automatically as part of each application evaluation process.

Using the trial design method, the tested varieties in the same ecological zone are automatically grouped according to the configured trial methods. For instance, since no more than 16 varieties can be included in each group, if there are 60 varieties for testing they will be randomly divided into four groups. The CK will be added automatically to each group, and each group will be assigned to specific test locations. After the test scheme design is completed, the system will automatically generate the test tasks which each group will need to perform at each test location.

**Figure 8:**
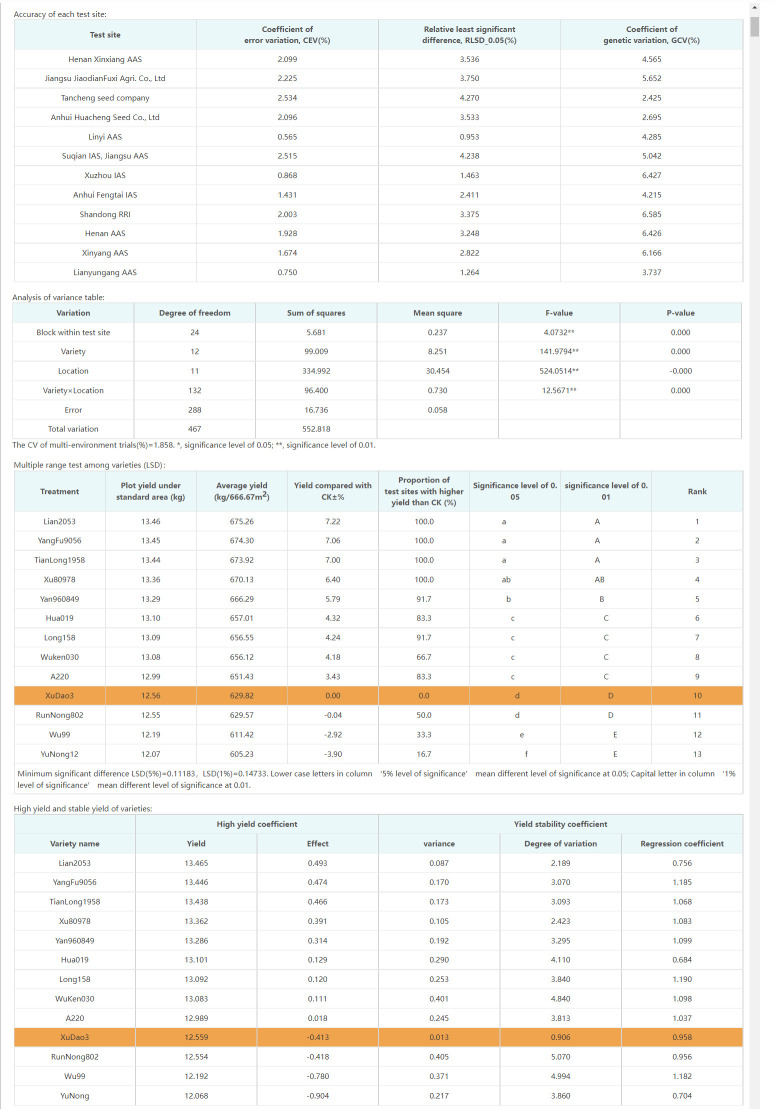
Data analysis interface provided by VTP.

**Figure 9:**
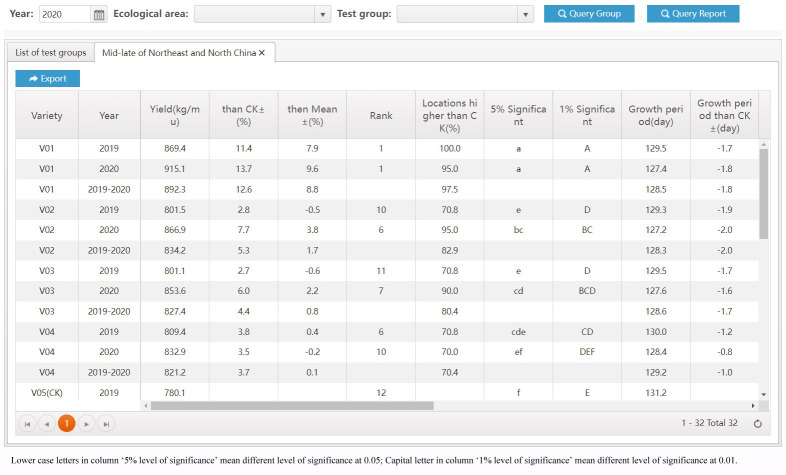
Variety test evaluation results.

**Figure 10:**
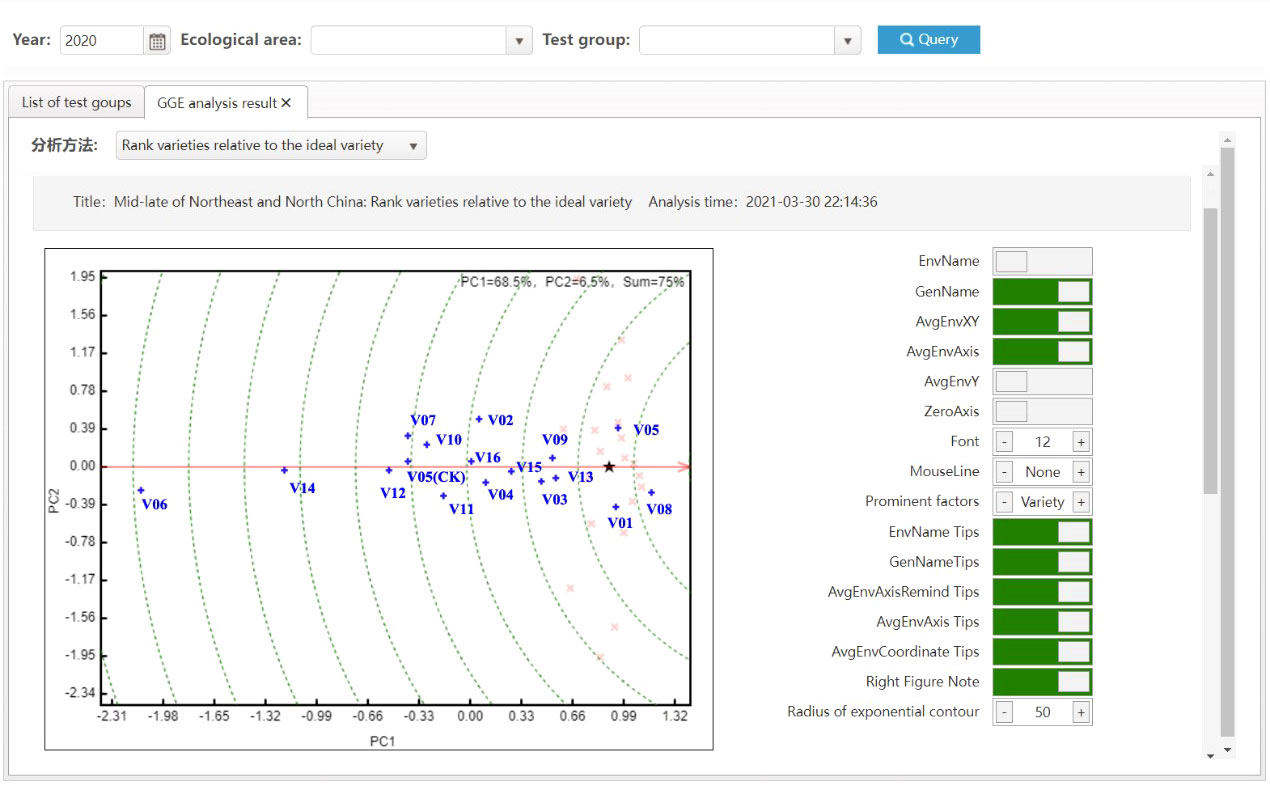
Ideal varieties analysis with GGE.

The most significant improvement in variety testing management efficiency is brought about by the streamlined data processing and analysis activities. The statistical analyses required for variety testing are complicated and require professional statistical knowledge. With the help of the VTP, managers can complete the test design and then process and analyze data easily and quickly. A data processing method, such as extracting the average, maximum, interval values, etc., can be assigned to each trait. Thus, multi-point test data of multiple years of trials can be analyzed automatically through only a few clicks, the analysis results is shown in **Figure 8**. In addition, after the test has been completed, the quality of each test location’s and each variety’s data needs to be summarized and analyzed, including extracting errors from missing data, error variation coefficients, variance statistics, as well as high yield and stable yield analysis. As shown in **Figure 9**, the yield increase and decrease, ranking, significance and other data of varieties relative to the CK can be obtained in real-time through the system. Meanwhile, the system also provides various visual analysis methods, as it has a built-in GGE double plot analysis method, including an ideal location ranking map, suitable planting area division map, ideal variety ranking map, etc., as shown in **Figure 10**.

The system also provides the function of generating test reports with one click. Once a test report template has been configured, the system can automatically generate objective descriptions of varieties, as well as relevant diagrams and tables according to the template. Before the VTP’s development, these test reports were created by the test directors manually, which was time-consuming and laborious. Now it only takes thirty minutes to generate a report automatically, and the test directors only need to add their own remarks on the evaluation of the trial.

### 3.4 Improvement in the quality of variety testing data

**Figure 11:**
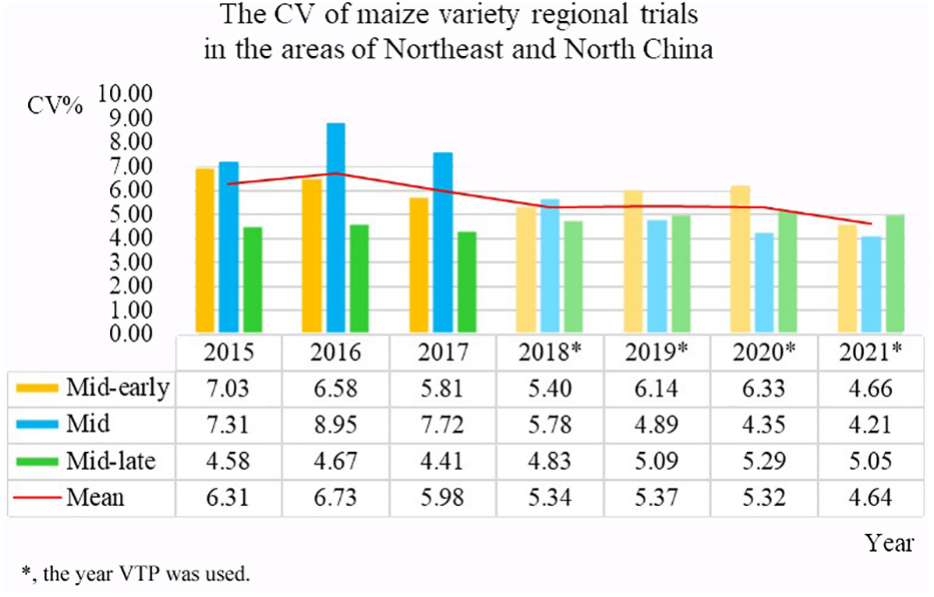
CV of variety regional tests in the areas of Northeast and North China.

Establishing a unified data standard and reducing human data entry errors will help ensure the quality of data obtained from the source. The CV is an important index that can be used to measure the quality of test data (Yan, 2014). In order to evaluate the effect of data quality control after the implementation of the VTP, the data error coefficients of variation of more than 50 test locations in three ecological zones in Northeast and North China, which was the main maize planting area in China from 2015 to 2021, were compared. The VTP had not been implemented during the 2015-2017 variety tests; it was used for tests conducted from 2018 to 2021. In **Figure 11**, the years in which the VTP was used to process data are marked using asterisks. The average CV of the test locations in 2015 was 6.31, while by 2021, it had dropped to 4.64. This represents a significant decline in error rates observed after the adoption of the VTP for the regional testing and shows that the VTP plays an important role in the quality control of test data.

### 3.5 Standard phenotypic database

**Figure 12:**
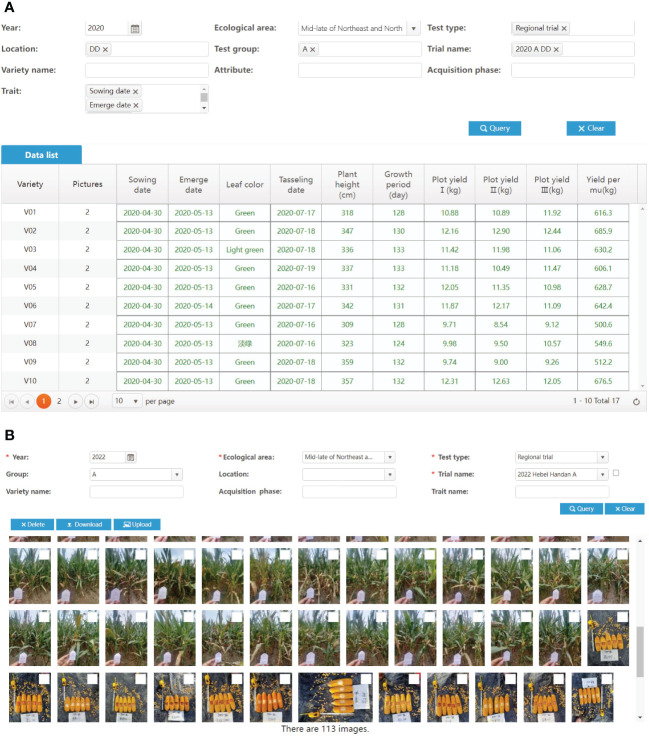
Test data recorded during variety testing. **(A)** Traits data. **(B)** Variety Images.

The unification of crop characteristic standards in the VTP lays the foundation for establishing a standard phenotypic database. By using the app or characteristic records, it is ensured that the data are entered in strict accordance with the standards. As shown in **Figure 12**, data from different test locations have the same trait descriptions. Therefore, a standardized phenotypic database can be formed by aggregating multi-location variety characteristic data for many years. Twenty thousand varieties have been managed using the VTP, with 26 million characteristic data entries and 350 thousand images. These data are stored in an Oracle 11g relational database using the EAV data model. The pictures collected by VTP are mainly RGB pictures of varieties, such as field seedlings, plants, ears, diseases and pests.

## 4 Discussion

Information systems have become an effective tool for breeding research and management (Zhao et al., 2022). The adoption of the VTP has resulted in a significant improvement of variety testing efficiency. Before VTP’s implementation, the variety test data were saved on paper and spreadsheets. The results of the practical applications reported in this paper confirm that an effective management tool is essential, as it can not only provide convenience for plant researchers, but also let more growers access variety information. The VTP provides users with a set of efficient test management tools, and some algorithms have been developed to compare results between the different tests and field conditions to discover new leads.

With the VTP, the standardization of the testing process and the quality of data are paid more attention. The traits to be collected may be different for each variety test in different programs, and the lack of unified descriptions can seriously hinder the exchange and reuse of data (Ćwiek-Kupczyńska et al., 2016). The analysis of these test data necessitates the storage of information in a standardized manner, preferentially in relational databases (Köhl et al., 2008). Moreover, it has been recognized that a lack of integration between genetic resources, characteristics and breeding, evaluation, and utilization data is a restrictive factor in the development of knowledge-intensive crop improvement plans (McLaren et al., 2005). In addition, the standardized management of the test process guarantees the generation of standardized and high-quality data. In addition, whereas past work on the subject was mainly focused on the management of test data through various software packages such as PhenoApp (Rckel et al., 2022) or Phenotyper (Köhl and Gremmels, 2015), little attention has been paid to the testing business process. The VTP can realize the standardized management of the whole variety testing process while supporting the customization of collected traits for each test program, including the characteristics to be recorded and their processing methods.

These phenotypic data are crucial for the development of multi-omics analysis. In multi-omics research, a large number of genotype data have been produced, but phenotypic data are relatively scarce (Zamir, 2013). In particular, the raw data generated from the phenotypic research programs conducted each year are rarely publicly available. For a long time, the major bottleneck to intelligent data integration and utilization has been the finding, extraction, preparation and management of the data (McLaren et al., 2005). Consequently, several international crop phenotype databases have been established, such as the International Crop Information System (McLaren et al., 2005) or the Online Farm Trials database (Walters and Light, 2021). In China, there is no relatively complete variety phenotype database. Through the application of VTP, a standardized and authoritative phenotypic database covering five crops was generated.

In addition, VTP, which has been implemented as a SaaS architecture, can be used to manage various independent testing programs, and especially for the self-organized tests of combination and green channels, whose personnel and infrastructure resources are relatively limited. Some large companies have developed trial management systems, but these are not publicly available. Other systems such as Phytotracker (Nieuwland et al., 2012) are used in only few research facilities, and have been designed for facilitating the task of keeping records of growing plants, seed stocks, and plasmids in molecular genetics labs. The integrated platform based on SaaS presented in this study is fully customizable and can potentially expand the use of real-time trial management tools for testers.

## 5 Conclusions

In this study, we reported a variety test platform, the VTP, which can be used to manage the whole workflow of multi-crop variety tests. First, this integrated data management system manages the whole test workflow and provides many functions which can help users carry out testing efficiently, such as test design, data acquisition and statistical analysis. Moreover, the obtained data quality can be significantly improved by formulating data element standards and standardizing the test process. Additionally, the VTP can be deployed in the cloud in available high-performance computing nodes so that the system can provide variety test management services to more small and medium-sized breeding organizations. Trial management and data analysis can be realized conveniently using a web-based interface. Thus, VTP not only provides an efficient tool for variety testing, but has also allowed the creation of a standardized and authoritative phenotype database. To our knowledge, this is the largest phenotypic database in China. In summary, we are thus confident that the platform can make variety testing more efficient and generate a reliable database suitable for meta-analysis in multi-omics breeding and variety development projects. With the further application of computer vision technology in variety testing, more phenotype data will be generated, and it is essential that these data are managed efficiently (Xing et al., 2022). With these standardized data, the construction of variety knowledge maps and intelligent analysis models combined with meteorological environment and gene data in the future will allow the development of intelligent decision support systems for variety promotion and the correlation analysis between phenotypes and genotypes.

## Data availability statement

The original contributions presented in the study are included in the article/Supplementary Materials. Further inquiries can be directed to the corresponding authors.

## Author contributions

FY and ZL conceived this research and wrote the manuscript. KW, JQ, YW designed and discussed the content of this study. XW, QZ, XZ, YH, SP, SW, QiZ developed the system. SY provided the basic dataset. All authors contributed to the article and approved the submitted version.
